# Anxiety as a risk factor for school absenteeism: what differentiates anxious school attenders from non-attenders?

**DOI:** 10.1186/1744-859X-12-25

**Published:** 2013-07-25

**Authors:** Jo Magne Ingul, Hans M Nordahl

**Affiliations:** 1 Department of Child and Adolescent Psychiatry, Levanger Hospital, Nord-Trøndelag Health Trust, Kirkegt 2, 7600 Levanger, Norway; 2 Department of Psychology, Norwegian University of Science and Technology (NTNU), Trondheim, Norway; 3 Department of Psychiatry, Levanger Hospital, Nord-Trøndelag Health Trust, Lavanger, Norway

**Keywords:** School absenteeism, Anxiety, Depression, Neuroticism, Behavioural problems, Risk factors

## Abstract

**Background:**

Anxiety is a major risk factor for problematic school absenteeism. However, most anxious students attend school. What differentiates anxious attenders from non-attenders?

**Method:**

High school students (*N* = 865) were assigned to groups based on anxiety and absenteeism scores. These groups were then tested for differences in risk factor profiles using discriminant analysis.

**Results:**

Anxious school attenders were less affected by negative personality traits, total number of risk factors, social anxiety, panic, and behavioural and family problems. They also displayed greater resilience.

**Conclusions:**

This study indicates that the risk for problematic school absenteeism increases as the number of risk factors aggregate and that treatment for anxious school refusal should be based on a profile of the individual's risk factors.

## Background

Absenteeism in high school is a serious public issue. Problematic absence exceeds the incidence of major childhood behavioural disorders and has been shown to be a major risk factor for dropping out, unemployment, economic deprivation, suicide attempts, psychiatric disorders, and substance abuse as children grow [1]. Many studies have shown that anxiety and anxiety disorders are related to problematic school absenteeism. Kearney and Albano [2] reported that the most common diagnoses in a group of 143 children 5 to 17 years old with problematic school absenteeism were separation anxiety disorder, generalized anxiety disorder, social phobia, oppositional defiant disorder (ODD), and depression. Egger et al. [3] found that approximately two out of three school refusers in a community sample met criteria for a psychiatric disorder, with anxiety disorders, depression, and ODD being the most common. Richards and Hadwin [4] explored the relationship between trait anxiety and school attendance and found that elevated trait anxiety was associated with higher absenteeism, but that this relationship could be partially explained by motivational factors. In a retrospective study [5], adults with anxiety disorders (*n* = 201) were found to report leaving school prematurely due to anxiety problems in 49% of the cases. Patients who left school early were more likely to have had a lifetime diagnosis of social phobia, a history of substance use, and a greater number of lifetime diagnoses. These and other studies clearly indicate that anxiety symptoms and anxiety disorders, especially social phobia, are associated with higher rates of school absenteeism [6-8] and also indicate that there are usually comorbid psychiatric and social problems.

Recent research with high school students suggests that anxiety alone does not predict problematic school absenteeism [9]. As in the previously mentioned studies, a study by Ingul et al. [9] showed that anxiety problems were prevalent in high-absence groups, but it also showed that many anxious students attend school. Egger et al. [3] reported that 12.5% (81) of a sample of school refusers had anxiety disorders, indicating that anxiety disorder is a major risk factor for school refusal. However, 12.4% (176) of the pupils in the nonschool-refuser group also had anxiety disorders. This implies that although anxiety is a risk factor for problematic school absenteeism, it is neither necessary nor sufficient to explain the problem, as many anxious pupils attend school, and many pupils are absent from school without being anxious. To explain problematic school absenteeism, we must consider the co-occurrence of other psychiatric, somatic, or social problems in addition to anxiety problems; studies have indicated that risk factors seem to aggregate over time and eventually lead to problematic school absenteeism [10-12].

In searching for additional factors, Egger et al. [3] found that in addition to psychiatric problems, school refusers reported sleep difficulties, higher rates of fears/worries, and somatic complaints (headaches and stomachaches), poorer peer relations, and lower socio-economic status (poverty, single parent household, parents treated for mental health problems). Lounsbury et al. [13] found that absenteeism is predicted by personality traits, including the ‘big five’ personality traits, implying that there might be a dispositional tendency towards school absenteeism, which was called ‘absence proneness’ [13]. However, a problem in this research area is related to terminology. Different researchers use different terms for school absenteeism (truancy, school refusal, and school phobia), others define the same concepts differently, and some researchers use different terms interchangeably [1]. Thus, making comparisons between studies are difficult [14].

In this study, we have focused on the overarching concept of school absenteeism or attendance. To our knowledge, no study has examined which factors differentiate between anxious school attenders and anxious non-attenders. The information gained from such a study might be useful in understanding the processes that lead to school absenteeism and thus help improve both treatment and prevention strategies. First, we hypothesized that the anxious non-attenders would show not only a higher incidence of comorbid problems, especially externalizing problems (behaviour and substance use), but also more symptoms of personality problems [13]. Second, because research indicates that high-absence adolescents have more negative life events, fewer close friends, school difficulties [3], lower resilience, greater likelihood of parents being out of work, and poorer health in their families [9], we expected that high-absence adolescents would have more psychosocial problems. Third, with respect to type of anxiety, we expected that the high-absence adolescents would report more social anxiety problems, as social phobia has been shown to be a risk factor for premature school withdrawal in adults [5]. We addressed these questions by examining groups with different levels of anxiety and absence in a sample of 865 adolescents, analysing which individual risk factors differentiated between high and low absence in groups of anxious students. Finally, we wanted to test for combinations of risk factors and their ability to distinguish between the different groups in the sample using multigroup discriminant analysis (MDA).

## Methods

### Participants

Adolescents from two high schools were recruited. In Norway, nine out of ten adolescents apply to and start high school [15]. Every adolescent has a right to attend high school. Most students finish in 3 years, but they are allowed up to 5 years to complete high school, which means that some students do not graduate until they are 21 years old. High schools try to integrate all adolescents, meaning that pupils with intellectual disabilities, learning disabilities, and physical and mental handicaps attend the same schools.

Eight hundred and sixty-five (84.4%) of the 1,025 eligible pupils participated in this study, 452 (52.3%) females and 413 (47.7%) males. They ranged in age from 16 to 21 years, with a mean age of 17.21 years (SD = 1.28). Seventy-five of the eligible pupils declined participation (7.4%), and the remaining non-participants were not present when the questionnaire was completed. There were no statistically significant differences between those who participated and those who did not, in gender (*χ*^2^ (1, 1025) = 0.41, *p* = 0.523), age (*M* = 17.18 (0.07), 17.22 (0.13), *t*(1,023) = −0.970, *p* = 0.332), or absence (*M* = 6.60 (0.29), 8.41 (0.88), *t* (195.26) = −1.95, *p* = 0.052).

### Procedure

After the regional ethics committee (Regional Komite for Medisinsk og Helsefaglig Forskningsetikk Midt-Norge no: 008–04) approved this research, we introduced the study to the school authorities in meetings and through written materials. Teachers distributed consent forms to the pupils, and those who wished to participate completed a questionnaire during school hours. Students who were not present when the questionnaire was distributed were asked to participate as soon as they were back in school, but no later than 2 weeks after the initial date. The teachers reported the absences for each student during the first term.

### Instruments

#### Anxiety

To measure anxiety symptoms, we used the Screen for Child Anxiety-Related Emotional Disorders (SCARED) [16]. The SCARED consists of 41 self-report items rated on a three-point scale. It has demonstrated very good psychometric properties in both community [17] and clinical [18] samples, with good internal consistency (Cronbach's *α* of 0.90–0.96 for the total score, and 0.74–0.86 for the subscales), test-retest reliability, and discriminant validity. The SCARED assesses the symptoms of four DSM-IV disorders, and it provides a total score and a school avoidance score. Cronbach's *α* for the SCARED in this sample was 0.94, with corresponding subscale coefficients of 0.87 for the panic/somatic syndrome scale, 0.88 for the generalized anxiety scale, 0.74 for the separation anxiety scale, 0.85 for the social phobia scale, and 0.62 for the school avoidance scale. The cut-off for differentiating between high- and normal-anxiety groups was based on the recommendations of Birmaher et al. [18] who used a score of 26 points as the cut-off for high anxiety.

#### Absenteeism

The schools reported absenteeism (in days and hours) for each participant. We divided the students into two groups based on their absence scores: the high-absence group missed 13.5 or more days, and the low- or normal-absence group missed 0–13.49 days. The cut-off for the high-absence group is consistent with Kearney's [14] criteria for problematic absence.

#### Depression

The short version of the Mood and Feeling Questionnaire (SMFQ) contains 13 items, and answers are given on a three-point scale. Higher scores indicate more severe depressive symptomatology [19]. The SMFQ has adequate reliability (Cronbach's *α* of 0.85) [20], and its criterion validity has been established using the Kiddie SADS (sensitivity 59.2% and specificity 89.7%) [21]. The psychometric properties of the SMFQ are acceptable [22]. In this study, the SMFQ had a Cronbach's *α* of .88, indicating high internal consistency.

#### Personality

We assessed personality problems with the Iowa Personality Disorders Screen (IPDS), which has shown good sensitivity and specificity [23]. In adolescents, the IPDS reaches a maximum hit rate (83.5%) and provides the best balance between positive and negative predictive powers (i.e. sensitivity 69.4% and specificity 91%) with a subset of items (1, 3–8) and a cut-off at two points [24]. The IPDS contains 11 items that correspond to diagnostic criteria of the DSM-IV for personality disorders [23,24].

#### Externalizing problems

The Strengths and Difficulties Questionnaire (SDQ) was used to measure externalizing problems. The SDQ is a behavioural screening instrument for symptoms and positive attributes [25]. It has 25 items, divided into five subscales (emotional symptoms, conduct problems, hyperactivity/inattention, prosocial behaviour, and peer problems). In a large community sample, Goodman [26] reported a mean internal consistency of 0.73 on all subscales and the total problem score. In this study, we obtained a Cronbach's *α* of 0.72 for the SDQ scale, with corresponding subscale coefficients of 0.58 for conduct problems and 0.68 for hyperactivity/inattention.

#### Substance abuse

Students were asked if they had ever used illegal substances (such as cannabis and other narcotics), and if so, how many times. The questionnaire also solicited information about the frequency and quantity of the students' alcohol consumption.

#### Resilience

We used the Resilience Scale for Adolescents (READ; [27]) to measure resilience. The 28 items on the READ use a five-point Likert scale response format. Higher scores indicate greater resilience. The scale has adequate psychometric properties (Cronbach's *α* of 0.94 for the total score) [27], and a correlational study indicated that it has a predictive power for anxiety and depression [28]. In this study, we obtained a Cronbach's *α* of 0.94.

#### Significant life events (SLE)

Participants reported the following 12 stressful life events: death in family, death of close friend, serious illness or injury, family divorce or separation, becoming socially distant from the family or a close friend, a break-up with boyfriend or girlfriend, problems at school, alcohol consumption, changing school, and experiencing personal violence. All items had a yes-or-no response format. The SLE score was obtained by summing the number of stressful life events reported. The SLE life events are part of the Life Events Scale of Holmes and Rahe [29] and the Life Events Checklist [30]. These tests contain a selection of severe life events that are frequently reported by adolescents (e.g. [31,32]).

#### School factors

To measure factors related to school, we constructed several categorical items concerning educational programmes, relationship with homeroom teachers, pupils' perceptions of how they were treated and respected at school, feelings of being safe in school, presence of learning difficulties, and experiences of being bullied in school. All of these factors have previously been identified as school-related risk factors for absenteeism [9].

#### Demographic variables

We also solicited information about the students' gender, parental education and occupational status, whether or not they lived with their parents, friendships (number of close friends), participation in leisure time activities, and self-reported physical health and chronic illness.

#### Psychiatric severity

Participants answered questions about symptoms of depression, personality problems, behavioural problems, hyperactivity problems, and substance abuse problems. The psychiatric severity score was obtained by summing the number of scales in which the student scored above the cut-off.

### Statistical analysis

We used independent sample *t* tests and chi-square tests to see if there were differences in risk factors and demographic variables between the high-absence/high-anxiety and low-absence/high-anxiety students. Analyses were run using SPSS version 18.

We used MDA to identify combinations of risk factors that could distinguish the anxiety by absence groups. Given the multivariate nature of interactions between the risk factors and the dependent variable, MDA is an appropriate way to analyse this type of data. It combines independent variables that classify groups, and it is appropriate for determining the best indicators of separation between groups. MDA is based on factor analytic methods that identify sets of variables that are powerful in discriminating between groups of subjects on a data-driven basis. MDA is typically a one-way analysis, and no problems are posed by unequal sample sizes in groups. The sample size in the different groups provided adequate statistical power (81.4%) in the current analysis [33].

## Results

We divided the 865 students into four groups based on their absence and anxiety scores. High- and normal-anxiety groups were formed using the recommended cut-off for the SCARED [18], and high- and normal-absence groups were formed based on Kearney's [14] criteria for problematic absence. Table 1 shows the number of males and females in each group, as well as their mean age, anxiety, and absence scores.

**Table 1:** Age, sex, absence, and anxiety in the four groups of students and total sample

**Group**	**Sex**	**Age**	**Absence in days**	**SCARED**
	**(% female)**	**( *M *, SD)**	**( *M *, SD)**	**( *M *, SD)**
High anxiety, high absence (*N* = 21)	85.7	17.57 (1.50)	25.08 (12.17)	40.00 (13.77)
High anxiety, low absence (*N* = 73)	79.5	17.45 (1.58)	5.74 (4.17)	34.62 (19.84)
Normal anxiety, high absence (*N* = 80)	52.5	17.59 (1.54)	22.94 (12.14)	9.97 (6.86)
Normal anxiety, low absence (*N* = 636)	48.0	17.11 (1.14)	3.86 (3.34)	8.29 (6.20)
Total sample (*N* = 865)	52.3	17.21 (1.28)	6.49 (8.32)	11.66 (11.15)

### Psychiatric severity and comorbidity

As predicted, the analysis showed that relative to the high-anxiety/low-absence group, the high-anxiety/high-absence group had more behavioural problems (*t* (92) = 2.39, *p* < 0.05), greater psychiatric severity (*t* (90) = 2.53, *p* < 0.05), and more frequent use of narcotics (*χ*^2^ (1) = 3.91, *p* < 0.05). However, these groups differ neither with respect to personality problems, alcohol, or cannabis use nor with respect to depressive symptoms (see Table 2).

**Table 2:** Comorbidity and psychiatric severity in high- and low-absent anxious students

**Variable**	**High absence**	**Low absence**			**Significance**	**Whole sample**^**a**^
	**high anxiety**	**high anxiety**				**(*N *= 865)**
	**(*N *= 21)**	**(*N *= 73)**	***t *Value ( *df *)**	***χ***^**2**^**( *df *)**		
Psychiatric severity	3.33 (1.20)	2.63 (1.09)	2.53 (90)		0.013	0.76 (1.35)
SDQ behavioural problems	3.23 (1.64)	2.23 (1.70)	2.39 (92)		0.019	1.75 (1.46)
Personality traits	4.71 (2.45)	4.56 (2.40)	0.252 (90)		0.802	1.98 (2.03)
SDQ relational problems	2.81 (1.69)	3.43 (2.21)	−1.18 (92)		0.241	1.77 (1.58)
SMFQ	12.05 (4.61)	10.01 (4.55)	1.80 (92)		0.075	4.95 (4.43)
Categorical variables						
Drinking alcohol at least every week (% yes)	9.52	25.58		0.582 (1)	0.445	7.7
Tried cannabis (% yes)	30	12.33		3.62 (1)	0.057	8.1
Tried other narcotics (% yes)	25	8.57		3.91 (1)	0.048	3.3

^**a**^ The whole sample is included for comparison.

### Psychosocial problems

Psychosocial problems were analysed using independent sample *t* tests and chi-square tests. The analysis showed that the two groups did not differ with respect to negative life events, resilience, family work, economy, or chronic health problems. However, students in the high-absence/high-anxiety group were more likely to perceive their own health as bad (*χ*^2^ (1) = 4.28, *p* < 0.05) and reported having fewer close friends (*t* (82.53) = −3.66, *p* < 0.001), but they also felt more respected (*χ*^2^ (1) = 4.89, *p* < 0.05 and less bullied at school (*χ*^2^ (1) = 4.21, *p* < 0.05). See Table 3.

**Table 3:** Psychosocial problems in high- and low-absent anxious students

**Variable**	**High absence**	**Low absence**			**Significance**	**Whole sample**^**a**^
	**high anxiety**	**high anxiety**				**(*N *= 865)**
	**(*N *= 21)**	**(*N *= 73)**	***t *Value ( *df *)**	***χ***^**2 **^**( *df *)**		
Negative life events	4.32 (2.58)	3.25 (2.39)	1.70 (86)		0.093	1.88 (1.87)
Resilience	3.24 (0.76)	3.50 (0.61)	−1.54 (80)		0.127	3.99 (0.63)
No. of close friends	4.04 (1.35)	4.76 (0.54)	−3.66 (82.53)		0.000	4.62 (0.85)
Family economy	3.29 (1.52)	2.88 (1.16)	1.14 (27.17)		0.263	2.54 (1.11)
Categorical variables						
Father working (% yes)	87.5	81.8		0.29 (1)	0.588	92.4
Mother working (% yes)	68.8	68.1		0.002 (1)	0.961	82.5
Being bullied at school (% yes)	10	35.7		4.89 (1)	0.027	8.4
Feeling safe in school (% yes)	28.57	36.62		0.166 (1)	0.683	89.8
Treated with respect in school (% yes)	95.20	81.94		4.21 (1)	0.040	94.60
Chronic illness (% yes)	42.86	35.62		0.589 (1)	0.443	30.6
Perception of own health (% bad)	66.67	41.10		4.28 (1)	0.038	15.1

^**a**^The whole sample is included for comparison.

### Type of anxiety

We used independent sample *t* tests to examine differences in type of anxiety between the two groups. This showed that as predicted, the high-anxiety/high-absence group had higher social anxiety scores (*t* (92) = 2.15, *p* < 0.05), and they also reported more symptoms of panic/somatic syndrome (*t* (92) = 2.08, *p* < 0.05) on the SCARED than did the high-anxiety/low-absence group. This indicates that the type of anxiety differentiates these two groups (see Table 4).

**Table 4:** Type of anxiety in high- and low-absent anxious students

**Variable**	**High absence**	**Low absence**	***t *Value ( *df *)**	**Significance**	**Whole sample**^**a**^
	**high anxiety**	**high anxiety**			**(*N *= 865)**
	**(*N *= 21)**	**(*N *= 73)**			
Social anxiety	9.24 (3.53)	7.59 (2.96)	2.15 (92)	0.034	3.32 (3.05)
Panic/somatic syndrome	12.05 (7.44)	9.14 (5.06)	2.075 (92)	0.041	2.68 (3.65)
Generalized anxiety	11.19 (3.80)	10.58 (3.14)	0.754 (92)	0.453	3.57 (3.79)
Separation anxiety	4.29 (3.05)	4.58 (2.58)	−0.446 (91)	0.657	1.20 (1.91)

^a^The whole sample is included for comparison.

### Combination of risk factors: multigroup discriminant analysis

We used MDA to test whether the different combinations of risk factors could distinguish between the four groups. All of the risk factors (except the anxiety measures) were used as predictors, and the dependent variables were the four anxiety (high and low) by absence groups. Three significant discriminant functions were identified: function 1 (Wilks' lambda (*Λ*) = 0.156, *χ*^2^ (99) = 653.86, *p* < 0.001), function 2 (Wilks' *Λ* = 0.633, *χ*^2^ (64) = 170.96, *p* < 0.001), and function 3 (Wilks' *Λ* = 0.862, *χ*^2^ (29) = 55.40, *p* < 0.01).

**Figure 1:**
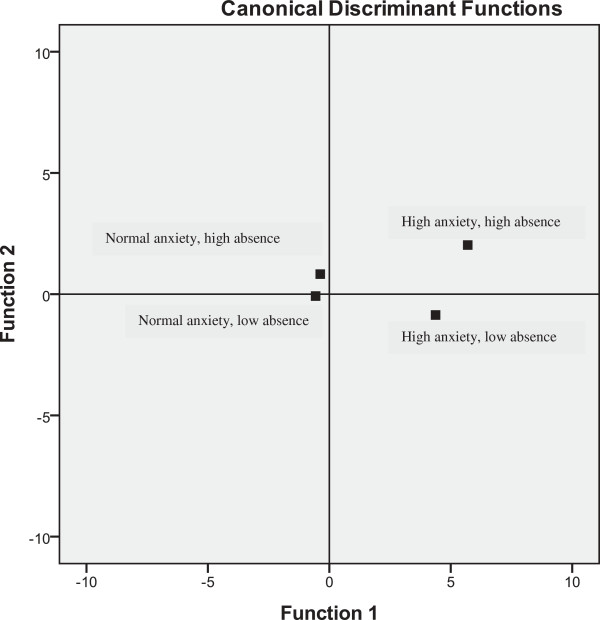
**Plots of four group centroids on two discriminant functions derived from risk factors for school absenteeism.** Function 1 represents high versus low levels of psychiatric problems, negative personality traits, and relational problems. Function 2 represents high and low levels of substance abuse and behavioural problems.

Table 5 shows the correlation matrices between the risk factors and each of the three functions. The canonical correlations for the three functions were 0.87, 0.52, and 0.37, respectively. The three discriminant functions accounted for 85.4% (function 1), 10.1% (function 2), and 4.5% (function 3) of the between-group variability. Function 1 contrasted high and low levels of psychiatric problems, characterized by negative personality traits and relational problems. Students with low scores on this function had fewer negative personality traits and few or no relational problems. Function 2 contrasted high and low levels of substance abuse and behavioural problems. Students with high scores on function 2 had more behavioural problems and more substance abuse problems. Function 3 contrasted high and low levels of resilience, characterized by participation in leisure time activities and exercise. Group centroids plotted on a perceptual map provide a visual representation of the differences between the groups on functions 1 and 2 (see Figure 1). A univariate ANOVA followed by *post hoc* tests (Fisher's LSD) showed that function 1 differentiated between all four groups (*p* < 0.001), function 2 differentiated between all groups except the high-absence groups (*p* < 0.001), and function 3 differentiated between all groups except the low-absence groups (*p* < 0.001). With respect to the two high-anxiety groups (high vs. low absence), the *post hoc* analysis showed that the high-anxiety/high-absence group scored significantly higher than the high-anxiety/low-absence group on both function 1 (*M* = 7.82, SD = 2.81 vs. *M* = 5.65, SD = 2.56, *p* < 0.001) and function 2 (*M* = 1.23, SD = 2.56 vs. *M* = −0.64, SD = 2.02, *p* < 0.001). On function 3, the scores were reversed; here, the high-anxiety/low-absence group scored significantly higher than the high-anxiety/high-absence group (*M* = 0.81, SD = 1.63 vs. *M* = −0.72, SD = 1.32, *p* < 0.01). Using the classification procedure for the full sample (*N* = 865), 73% of the students were correctly classified. A further analysis of the classification procedure showed that it was mainly between the low-anxiety/low-absence and low-anxiety/high-absence groups that the procedure made misclassifications (28.2% of these cases were misclassified).

**Table 5:** Correlation between the discriminant functions of risk factors and each risk factor for the groups

**Predictor**	**Discriminant function**		
	**Function 1**	**Function 2**	**Function 3**
Psychiatric severity	*0.872*^a^	−0.070	−0.169
Personality traits	*0.318*^a^	0.063	−0.120
SMFQ	*0.308*^a^	0.125	−0.053
SDQ relational problems	*0.239*^a^	−0.164	−0.108
Safe in school	*−0.236*^a^	0.153	0.070
Resent going to school	0.190^a^	0.082	−0.084
Perception of own health	−0.181^a^	−0.146	0.129
Being bullied	0.163^a^	−0.059	0.027
Chronic illness	0.120^a^	−0.038	0.029
Used narcotics	0.168	*0.546*^a^	0.439
Used cannabis	0.116	*0.301*^a^	0.093
Mother working	−0.021	*−0.279*^a^	0.098
SDQ behavioural problems	0.138	*0.256*^a^	0.022
No. of close friends	0.127	*0.242*^a^	0.236
Negative life events	0.191	*0.222*^a^	−0.094
Living with parents	−0.161	*−0.215*^a^	0.064
Drinking least every week	0.077	0.178^a^	−.113
Mother's education	−0.063	−0.159^a^	0.076
Contact with teacher	−0.043	0.135^a^	0.070
Learning difficulties	−0.037	0.126^a^	0.099
School size	0.013	0.063^a^	−.038
Leisure time activities	−0.088	−0.134	*0.370*^a^
Age	0.029	0.160	*−.319*^a^
SDQ hyperactivity	0.120	0.177	*−0.305*^a^
Treated with respect at school	−0.084	−0.096	*0.303*^a^
Exercising	−0.056	−0.208	*0.231*^a^
Resilience	−0.184	−0.030	*0.207*^a^
Father working	−0.101	−0.165	0.187^a^
Sex	0.090	0.019	0.146^a^
Family economy	0.039	−0.068	0.100^a^
Father education	−0.039	0.032	0.078^a^
Canonical *R*	0.868	0.516	0.371
Eigenvalue	3.06	0.363	0.160

^a^Largest absolute correlation between each variable and any discriminant function. The highest loading items on each function are italicized.

## Discussion

The purpose of this study was to determine what differentiates between anxious students who attend school regularly and anxious students with high absenteeism rates. We found that type of anxiety (degree of social anxiety and panic symptoms), the presence or absence of behavioural and substance abuse problems, psychiatric severity, perception of own health, and number of close friends all discriminated between these two groups. There were also differences with respect to two school factors, namely extent of bullying and whether or not students felt that they were treated with respect at school. These factors are interesting, because the anxious students who attended school regularly reported experiencing more bullying and feeling less respect at school than the anxious students with high absenteeism rates. This probably reflects the fact that the regular attendees spent more time in (what they regarded as) a hostile school environment than the high-absentee students did. The MDA analysis indicated that psychiatric severity and negative personality traits are the most important differentiating factors, followed by comorbid behavioural problems and the students' degree of resilience.

These findings may have important implications. First, they suggest the extent of anxiety-related problems in schools, and they indicate that negative personality traits and relational problems are the most important risk factors for school absenteeism. Function 1 indicates that it is the total symptom burden and the presence of negative personality traits that differentiate between the two high-anxiety groups. Other studies in recent years have reached similar conclusions. Psychiatric comorbidity, low socio-economic status, and employment have been identified as individual predictors for drop-out, and research also indicates that risk factors aggregate over time, eventually leading to premature departure from school [10-12,34]. The findings from this and other studies indicate that some children may start school with individual risk factors that predispose them for absenteeism, such as anxiety, negative personality traits or negative parental attitudes towards school achievement, and school attendance. Over the years, developing psychopathology and/or learning disorders and an unsafe or poor school climate may further increase their chances for absenteeism. Function 1 also indicates that these students may have a tendency to interpret things in a negative way (they resent going to school, feel unsafe in school, and have a poorer perception of their own health). This may be a direct result of negative personality traits and may also reflect a tendency of high absentees to have more negative cognitions and to overgeneralize, as shown by Maric et al. [35].

There were several findings in this study regarding the difference between anxious attenders and non-attenders. The anxious school attenders were not as socially anxious as the anxious non-attenders, and they also had more friends. In addition, they reported being less frightened by the somatic symptoms of anxiety. These findings are supported by other studies [5,8]. Heyne et al. [8] treated anxious school refusers with the @-School Programme. At follow-up, adolescents diagnosed with a social phobia had significantly lower school attendance than adolescents with another anxiety disorder and adolescents who no longer met the criteria for an anxiety disorder. They also found that lonely adolescents (those who reported having no friends in their class) were the worst at follow-up, mirroring findings from this study.

Furthermore, reactions to feelings of being scared or anxious seem to differentiate the two groups. The more students experience symptoms of panic, the more likely they are to be absent from school. Students who score high on this factor fear the sensations of anxiety and harm they may bring. Mattis and Ollendick [36] suggest that adolescents who react with panic symptoms have learned to associate negative events with physical symptoms and experience intense alarm reactions with little sense of predictability or control over stressors. This leads to apprehension and avoidance of situations that set off these alarm reactions—in this instance, any school-related issues or situations.

In this study, anxious school attenders had lower rates of behavioural problems than the anxious non-attenders. Both in the single risk factor analysis and in the MDA analysis, behavioural problems and related phenomena such as substance abuse were strong differentiating factors between the high- and low-absence high-anxiety groups. Function 2 in the MDA analysis indicates that students with more behavioural problems and substance abuse also experienced other phenomena typically associated with behavioural problems at a greater rate, such as negative life events and indicators of low socio-economic status (mother not working, mother with low education, and living alone). Egger et al. [3] described a group of school refusers who met the criteria for both anxiety disorders and disruptive behaviour disorders. Relative to the other groups in the study, this mixed group had higher rates of absence, was younger at age of onset, and was less active in extracurricular activities. They also had fewer friends, lower socio-economic status, and parents who were more likely to be treated for mental health problems. Thus, members of the mixed group came from home environments that lacked the conditions for a safe and secure upbringing.

There are some limitations in the present study that need to be considered. From these data, we cannot say anything about the temporal relationships between the risk factors and school attendance. This means that no causal inferences can be drawn from the data. However, the aim of this study was to examine the prevalence, characteristics, and differences between anxious school attenders and anxious school non-attenders. Second, using cut-off scores to define group membership can be viewed as a weakness. Cut-off points have been criticized for being arbitrary, with the chance that minor changes in the cut-off could lead to completely different prevalence rates and characteristics. We recognized this risk and took care to base our cut-off points for both anxiety [18] and absence [14] on empirically derived thresholds. Third, the study does not contain any measure of cognitive function in the students, and this may have affected the results. Although lower cognitive functioning has been associated with higher absenteeism in some studies, most studies have generally supported the notion that children with high absenteeism are of average intelligence and display adequate academic achievements prior to their absenteeism [37]. Finally, the generalizability of the discriminant functions should be cross-validated to test the utility of the coefficients for other samples. This could be done by splitting a sample into two groups, deriving classifications in one group, and testing them on the other group. Another approach is to derive the classification functions from a sample at time one and retest them at time two [33]. However, neither of these approaches was feasible in the present study.

## Conclusions

With these limitations in mind, the main finding of this study is that relative to anxious non-attenders, anxious school attenders have fewer overall problems. Individually, they show fewer negative personality traits and social anxiety, and fewer panic symptoms and behavioural problems. They have fewer problems in their families, have friends, and in school. The findings in this study are important because they indicate that simply treating the anxiety problems of anxious school refusers may not be sufficient. In addition, clinicians should be especially aware of and target social isolation, behavioural problems, and family issues. On the other hand, the findings also indicate that building resilience and participation in prosocial activities could be beneficial and decrease the risk of absenteeism. Overall, these findings indicate that current treatments for anxious school refusal may be too narrow; we may need to broaden our approach and include modalities or components from other problem areas in our treatment manuals. The required dosages, sequencing, and effects of such interventions are as yet unknown and need to be documented.

## Abbreviations

ANOVA: Analysis of variance; IPDS: Iowa Personality Disorders Screen; MDA: Multigroup discriminant analysis; ODD: Oppositional defiant disorder; READ: Resilience Scale for Adolescents; SCARED: Screen for Anxiety Related Emotional Disorders; SDQ: Strengths and Difficulties Questionnaire; SMFQ: Short Mood and Feeling Questionnaire.

## Competing interests

The authors declare that they have no competing interests.

## Authors' contributions

JMI performed statistical analysis and prepared the draft. HMN supervised the data collection and helped draft the manuscript. Both authors read and approved the final manuscript.
